# EEG oscillatory patterns and classification of sequential compound limb motor imagery

**DOI:** 10.1186/s12984-016-0119-8

**Published:** 2016-01-28

**Authors:** Weibo Yi, Shuang Qiu, Kun Wang, Hongzhi Qi, Feng He, Peng Zhou, Lixin Zhang, Dong Ming

**Affiliations:** Department of Biomedical Engineering, College of Precision Instruments and Optoelectronics Engineering, Tianjin University, Tianjin, China; Tianjin Key Laboratory of Biomedical Detecting Techniques and Instruments, Tianjin University, Tianjin, China

**Keywords:** Sequential compound limb motor imagery, Event-related desynchronization, Phase locking value, Brain-computer interface, Support vector machine

## Abstract

**Background:**

A number of studies have been done on movement imagination of motor sequences with a single limb. However, brain oscillatory patterns induced by movement imagination of motor sequences involving multiple limbs have not been reported in recent years. The goal of the present study was to verify the feasibility of application of motor sequences involving multiple limbs to brain-computer interface (BCI) systems based on motor imagery (MI). The changes of EEG patterns and the inter-influence between movements associated with the imagination of motor sequences were also investigated.

**Methods:**

The experiment, where 12 healthy subjects participated, involved one motor sequence with a single limb and three kinds of motor sequences with two or three limbs. The activity involved mental simulation, imagining playing drums with two conditions (60 and 30 beats per minute for the first and second conditions, respectively).

**Results:**

Movement imagination of different limbs in the sequence contributed to time-variant event-related desynchronization (ERD) patterns within both mu and beta rhythms, which was more obvious for the second condition compared with the first condition. The ERD values of left/right hand imagery with prior hand imagery were significantly larger than those with prior foot imagery, while the phase locking values (PLVs) between central electrodes and the mesial frontocentral electrode of non-initial movement were significantly larger than those of the initial movement during imagination of motor sequences for both conditions. Classification results showed that the power spectral density (PSD) based method outperformed the multi-class common spatial patterns (multi-CSP) based method: The highest accuracies were 82.86 % and 91.43 %, and the mean values were 65 % and 74.14 % for the first and second conditions, respectively.

**Conclusions:**

This work implies that motor sequences involving multiple limbs can be utilized to build a multimodal classification paradigm in MI-based BCI systems, and that prior movement imagination can result in the changes of neural activities in motor areas during subsequent movement imagination in the process of limb switching.

## Background

Motor imagery (MI), defined as the mental rehearsal of a motor act without any overt motor output, can modify the neuronal activity in the primary sensorimotor areas in a manner which is quite similar to motor execution [1, 2]. MI-based brain-computer interface (BCI) systems could translate the subjective movement consciousness of the users without any inducing factors such as visual stimulus producing steady-state visual evoked potential (SSVEP) or event-related potential (ERP). This provides an alternative communication and control channel for patients with limited motor function to improve the quality of their lives [3]. Therefore, MI-based BCI systems play an important role in the field of rehabilitation engineering.

Since Jasper and Penfield’s discovery of brain oscillatory activity induced by MI [4], MI-based BCI systems have gone through several decades of development. However, a major problem is the limitation of control commands in contrast to other BCI paradigms. Most research on MI-based BCI systems have focused on analyzing EEG rhythms induced by simple limb motor imagery involving a single part of the limbs such as the left hand, the right hand or the foot [5, 6]. Pfurtscheller et al. studied the reactivity of EEG rhythms in association with the imagination of three kinds of limb movements (right hand, left hand, and foot) with the addition of tongue movement [7]. To implement a two-dimensional cursor movement between arbitrary positions, P300 potential was combined with MI (left and right hands) for controlling the horizontal and vertical movements of the cursor, respectively [8]. Additionally, MI was applied as a brain switch in a hybrid BCI system by detecting the post imagery beta event-related synchronization (ERS) of foot imagery to turn a four-step electrically driven hand orthosis with two flickering lights on and off to reduce the false positive rate during the resting period [9].

On the other hand, a significant amount of work have been reported on movement imagination of motor sequences with a single limb such as sequential finger-to-thumb opposition tasks [10–12], sequential dorsiflexion, and plantarflexion of the foot [13]. Lafleur et al. used positron emission tomography (PET) to measure and compare the dynamic changes in cerebral activity during the execution and imagination of sequential dorsiflexion and plantarflexion of the left ankle before and after practice [14]. A similar experiment was extended from the lower to the upper extremities to investigate the cerebral activations underlying the preparation and the execution periods associated with the actual and imagined movements of externally paced sequence of finger key press using EEG [15]. Moreover, Kranczioch et al. measured movement-related potentials to compare the functional similarity of the prediction mechanism during overt and covert action preparations of simple or complex sequential finger movements [16]. However, brain oscillatory patterns induced by sequential compound limb motor imagery (SCL-MI), particularly movement imaginations of motor sequences involving multiple limbs, have not been reported in recent years.

Limited choices of limb movement lead to the limitation of control commands in MI-based BCI systems. In addition, EEG patterns induced by simple limb MI are similar to those of sequential movement imagination of the same limb, and the latter case would activate the same functional area during sequential movement imagination of a single limb. In contrast with simple limb MI or sequential movement imagination of a single limb, different limbs are involved in SCL-MI, which can activate the neurons’ oscillation in different functional areas of the cerebral cortex within specific time intervals sequentially, and at the same time, is more in line with the normal behavior of humans. Furthermore, SCL-MI can achieve the output of multiple control instructions in MI-based BCI systems compared with the limited commands of simple limb MI.

In this study, we aimed to verify the feasibility of the application of SCL-MI to MI-based BCI systems. Additionally, the changes of EEG patterns and the inter-influence between movements in the motor sequences were also investigated. To this end, four kinds of sequential movement imagination were designed to simulate the movement of playing drums, involving one sequential movement imagination of a single limb and three kinds of SCL-MI based on the cooperation of two or three limbs. To test the separability of these four MI tasks, a multi-class common spatial patterns (multi-CSP) algorithm (successfully used in [17]) and power spectral density (PSD) were employed in feature extraction. Event-related spectral perturbation (ERSP) and phase synchronization analysis were applied to study the induced brain oscillatory patterns during SCL-MI.

## Methods

### Experimental procedure

The four motor sequences consist of three sub-movements. One sequential movement imagination of a single limb is [right hand]–[right hand]–[right hand] (RRR) and it represents imagining playing drums using the right hand according to the sequence. The three kinds of SCL-MI are [right hand]–[left hand]–[right hand] (RLR), [left hand]–[right foot]–[left hand] (LFL), and [right foot]–[right hand]–[left hand] (FRL) and they represent imagining playing drums using the corresponding limbs according to the sequence.

**Fig. 1:**
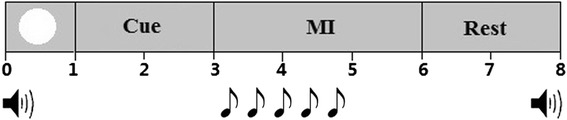
Experimental paradigm of the first condition. A white circle indicates the beginning of each trial, followed by a cue indicating the type of MI task. The subjects performed the indicated MI task for 3 s, and then rested for 2 s before the next trial. Two loudspeakers and the notes indicate the drum sounds during the whole experiment

**Fig. 2:**
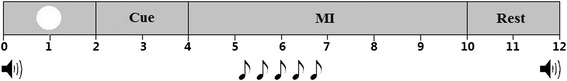
Experimental paradigm of the second condition. A white circle indicates the beginning of each trial, followed by a cue indicating the type of MI task. The subjects performed the indicated MI task for 6 s, and then rested for 2 s before the next trial. Two loudspeakers and the notes indicate the drum sounds during the whole experiment

Twelve right-handed healthy subjects (7 females and 5 males, 21–26 years old) participated in this experiment. The subjects sat in a chair at a 1-meter distance in front of a computer screen. There were two task conditions. The experimental paradigm of the first condition is shown as Fig. 1. Each trial (8 s) began with a white circle that appeared at the center of the monitor for 1 s. After the disappearance of the white circle, a character indication (‘[right hand]–[right hand]–[right hand]’, ‘[right hand]–[left hand]–[right hand]’, etc.) was presented on the screen for 2 s. During this phase, the subjects were instructed to mentally prepare and remember the task, while withholding any imagination. At the 3rd second, the cue disappeared and the screen turned black for 3 s. The participants were asked to immediately perform the indicated task once the screen turned black. MI tasks were performed kinesthetically rather than visually while avoiding any motion during imagination. At the 6th second, ‘Rest’ was presented for 2 s before the next trial. During the whole experiment, the subjects were asked to listen to the drums starting simultaneously with the appearance of the white circle (60 beats per minute), that is, there was a drum sound every second to maintain the self-rhythm of the imagination. Figure 2 shows the paradigm of the second condition (12 s), which is similar to the paradigm of the first condition, and accompanied by the drums (30 beats per minute) while the imagination phase lasts for 6 s. Three sub-movements in each motor sequence were performed sequentially, while a single sub-movement was performed once within a 1-s or 2-s time interval.

The experiments for both conditions were divided into seven sections, consisting of 40 trials each for the four kinds of sequential movement imagination (10 trials for each task in 1 section). The four MI tasks were presented in a randomized order. The intersection break lasted for about 5 to 10 min. In total, there were 280 trials (70 trials per class) in the study dataset.

Prior to recording, subjects were required to take three sessions for the training, consisting of five sections each (once a day) for both conditions for their familiarization and proper understanding of the tasks.

EEG data were recorded from 64 Ag/AgCl scalp electrodes placed according to the International 10/20 System referenced to nose and grounded prefrontal lobe. The EEG signals were acquired by a Neuroscan SynAmps2 amplifier whose sampling rate is 1000 Hz and the band-pass filtering range is 0.5–100 Hz. An additional 50-Hz notch filter was used during data acquisition. Thereafter, the original EEG signals were downsampled at 200 Hz. Before further analysis, the common average reference (CAR) was adopted during pre-processing.

The study was approved by the ethical committee of Tianjin University. All subjects signed their informed consent in advance.

### Event-related spectral perturbation

The event-related spectral perturbation (ERSP) method was used to inspect the spectral power changes of the induced EEG relative to the stimulus from the views of the time-frequency domain, which could supply more details about ERD/ERS patterns of different types of MI. The changes of event-related spectral power were analyzed with ERSP defined as follows:

1 $$ ERSP\left(f,t\right)=\frac{1}{n}{\displaystyle \sum_{k=1}^n\left({F}_k{\left(f,t\right)}^2\right)} $$

where *n* is the number of trials, and *F*_*k*_(*f*, *t*) is the spectral estimation of the *k*th trial at frequency *f* and time *t* [18, 19]. Short-time Fourier transform (STFT) was employed to perform time-frequency analysis of EEG data using a Hanning-tapered window in EEGLAB. The number of windows was set to 200 so that the window length is 256 points for the first condition and 512 points for the second condition. Baseline-normalized ERSP values (dB) were calculated from −3 to 5 s and from −4 to 8 s for the first and second conditions, respectively, relative to a baseline period (3 s before movement onset for the first condition and 4 s before movement onset for the second condition). In this study, the time-frequency ERD/ERS maps from three key electrode positions C3, Cz, and C4 were presented between 1 and 30 Hz for analysis because these three electrode positions are located over the right hand, the foot, and the left hand representation areas, respectively. The ERSP in the remainder of this paper refers to the baseline-normalized ERSP.

To investigate the influence of the prior sub-movement on the subsequent sub-movement, we measured the alpha ERD values of the second or third sub-movement imagination by integrating the ERSP values within the corresponding frequency bands and time intervals according to equation (2), as follows:

2 $$ ER{D}_{value}=\frac{1}{N}{\displaystyle \sum_{f\in F}{\displaystyle \sum_{t\in T}\left( ERSP\left(f,t\right)\right)}} $$

where *F* represents the ERD band and *T* represents the time interval (1 s or 2 s). *N* is the number of time-frequency bins in a selected rectangular area. The ERD values of the right and left hands were calculated for electrodes C3 and C4, respectively.

### Phase synchronization analysis

Phase synchronization is encountered in weakly interacting oscillator systems and it manifests through the occurrence of a relationship between the corresponding phases’ variables [20]. The degree of synchronization between two signals could be evaluated by the phase locking value (PLV), providing a reliable measurement of phase coupling [21, 22]. At first, the original EEG signals were band-pass filtered within a specific frequency band. Then, the instantaneous phases of the signals could be extracted by Hilbert transformation, and the difference of instantaneous phases corresponding to two different signals was defined as *Δφ*(*t*) = *φ*_*x*_(*t*) − *φ*_*y*_(*t*). After obtaining the relative phases, the degree of synchronization between any two signals was evaluated on a single trial basis using PLV, defined as

3 $$ PLV(t)=\left|\left\langle {e}^{j\varDelta \varphi (t)}\right\rangle \right| $$

where 〈 ⋅ 〉 denotes the temporal average over a time interval. Thereafter, the mean PLV was calculated by averaging all trials for each kind of movement imagination. In this way, the PLV ranges from 0 (no synchronization) to 1 (phase synchronization), providing an indication of the degree of interaction between the two underlying systems.

In this study, to investigate the involvement of the mesial frontocentral cortex during the imagination of sequential movement, phase synchronization was used to evaluate the underlying relationship between central electrodes (C3, Cz, and C4) and the mesial frontocentral electrode (Fcz) [23–25] during imagination of each sub-movement. The signals from these four electrodes were band-pass filtered between 8 and 13 Hz to focus on the EEG activities of the mu rhythm. PLVs were calculated according to equation (3) on three pairs of electrodes (C3-Fcz, Cz-Fcz, and C4-Fcz) for sub-movement imagination (within 1 or 2 s) in each sequence for further investigation, that is, studying the PLVs over C3-Fcz, Cz-Fcz, and C4-Fcz for the right hand, right foot, and left hand movement imaginations, respectively.

### Classification

#### Multi-class common spatial patterns based method

A common spatial patterns (CSP) algorithm has been widely used to extract the features of two MI classes based on multi-channel EEG information. Due to the property of CSP for binary situation, it has to be modified into a multi-class common spatial patterns (multi-CSP) algorithm to be suitable for the circumstance of multi-class MI tasks. Similar to the steps in [26], we first obtained the average covariance matrix *Σ*_*i*_ of each MI pattern, *i* ∈ {1, 2, 3, 4}. The whitening matrix can be obtained by

4 $$ P={\varLambda}^{\raisebox{1ex}{$-1$}\!\left/ \!\raisebox{-1ex}{$2$}\right.}{U}_0^T $$

where *U*_0_ is the matrix of eigenvectors and *Λ* is the diagonal matrix of eigenvalues from

5 $$ \varSigma ={\displaystyle \sum_{i=1}^4{\varSigma}_i}={U}_0\varLambda {U}_0^T $$

Thereafter, to acquire the spatial filter matrix relevant to the first class, we let ${\varSigma}_1^{\hbox{'}}={\displaystyle \sum_{i=2}^4{\varSigma}_i}$ according to the strategy of one-versus-rest, and if *Σ*_1_ and *Σ*_1_^'^ can be translated as

6 $$ {Y}_1=P{\varSigma}_1{P}^T\kern1em {Y}_1^{\hbox{'}}=P{\varSigma}_1^{\hbox{'}}{P}^T $$

then *Y*_1_ and *Y*_1_^'^ share common eigenvectors

7 $$ {Y}_1={U}_1{\varLambda}_1{U}_1^T\kern1em {Y}_1^{\hbox{'}}={U}_1{\varLambda}_1^{\hbox{'}}{U}_1^T $$

With the projection matrix *W*_1_ = *U*_1_^*T*^*P* consisting of spatial filters corresponding to the first class, the other three projection matrices could be obtained similarly.

The support vector machine (SVM) was adopted here for the classification of four kinds of sequential MI tasks. In this study, we used the LIBSVM software package to solve the multi-class classification problem. The multi-channel (64) EEG data were band-pass filtered between 8 and 30 Hz [26], including the whole MI period. By a tenfold cross-validation strategy, the training set served as the input of multi-CSP algorithm to achieve spatial filter matrices. For each direction of an MI task, only the eigenvectors corresponding to the first *l* eigenvalues could be used as spatial filters. The log variances of the spatially filtered EEG data were then used as the extracted features. The final classification accuracy $acc=\frac{1}{10}{\displaystyle \sum_{k=1}^{10}ac{c}^{(k)}}$ where *acc*^(*k*)^ is the classification accuracy of the testing set for the *k*th fold. The filter combination with the highest classification accuracy was the optimal combination.

#### Power spectral density based method

We extracted the power spectral density (PSD) as the feature for each MI task using the EEG data during imagination by sliding a short window in steps of 50 % overlaps, in which the PSD was calculated for 200 samples (1-s interval) shifted by 100 samples (0.5 s). Each segment was put into a PSD estimator using Burg's method (the order of an autoregressive model was 5). The sums of PSD in five frequency bands (delta: 0.5-4 Hz; theta: 4–8 Hz; alpha: 8–13 Hz; beta: 14–30 Hz; gamma: 30–50 Hz) were extracted for each segment, resulting in 5*m (*m* is the number of segments) features per channel for 62 channels (except HEO, VEO).

Support vector machine-recursive feature elimination (SVM-RFE), the most popular recursive feature-elimination algorithm, was applied to select the features for elimination. The RFE selection method is a recursive process that ranks features according to some measure of their importance [27]. However, in this study, the features coming from the same channel were regarded as a group; thus, we measured the importance of *N* channels. After constructing a final ranking of all channels, the features of the first *n* channels were taken as the input of SVM to obtain the corresponding classification accuracy using a tenfold cross-validation strategy. The channel combination with the highest classification accuracy was the optimal combination.

## Results

### Time-frequency analysis

**Fig. 3:**
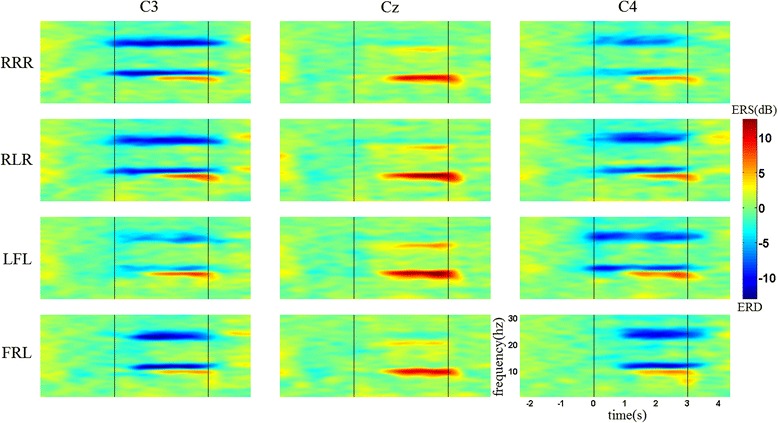
Examples of time-frequency maps for the first condition from one subject at three electrode locations. RRR, RLR, LFL, and FRL indicate [right hand]–[right hand]–[right hand], [right hand]–[left hand]–[right hand], [left hand]–[right foot]–[left hand], and [right foot]–[right hand]–[left hand], respectively. Blue represents ERD and red represents ERS. The first and second vertical lines represent the onset and end of MI, respectively. Time 0 represents the time point of cue offset

Figure 3 shows the time-frequency maps of the four MI tasks for the first condition from one subject at electrode positions C3, Cz, and C4. The maps present obvious ERD patterns in both mu (11–13 Hz) and beta (22–24 Hz) rhythms for all the MI tasks. However, the phenomenon of ERD changes over time is not clear. Taking a close look at the ERD patterns at electrode C4 during imagination of LFL, slightly weaker ERD can be found within the 2nd second. There are no significant differences between the ERD patterns at C3 and C4 for the sequences of RLR and FRL. Besides the induced mu and beta ERD, there are also long-lasting ERS patterns (around 10 Hz) observed starting from almost 1 s after the MI onset at all these electrodes.

**Fig. 4:**
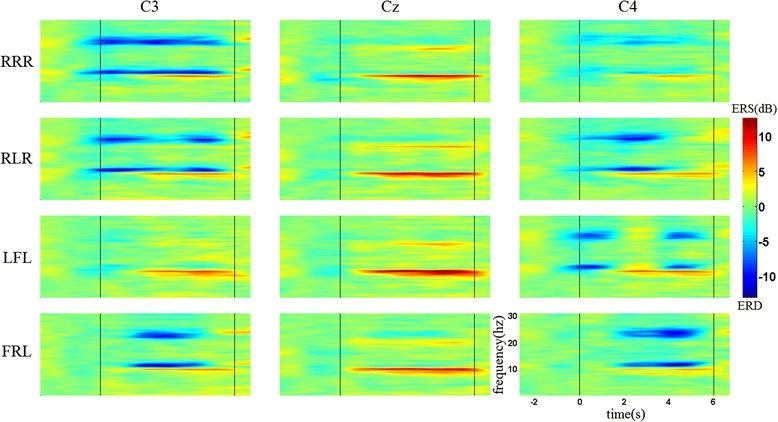
Examples of time-frequency maps for the second condition from the same subject at three electrode locations. RRR, RLR, LFL, and FRL indicate [right hand]–[right hand]–[right hand], [right hand]–[left hand]–[right hand], [left hand]–[right foot]–[left hand], and [right foot]–[right hand]–[left hand], respectively. Blue represents ERD and red represents ERS. The first and second vertical lines represent the onset and end of MI, respectively. Time 0 represents the time point of cue offset

Figure 4 shows the time-frequency maps of the four MI tasks for the second condition from the same subject at electrode positions C3, Cz, and C4. The maps show obvious ERD and ERS patterns within the same frequency bands as the first condition. In addition, the ERD patterns present better time-variant features, with apparent contralateral dominances for left/right hand imagery during the sequences. Compared with the sequence of RRR, quite different ERD patterns can be found for the other three sequences with discontinuous ERD over time at electrodes C3 and C4. ERD blocking is present at electrode C3, while the ERD feature reappears within the corresponding time interval at electrode C4 during the imagination of RLR. A similar phenomenon can be observed for the sequences of LFL and FRL. At the same time, no ERD feature induced by the sub-movement of the right foot is present at electrode Cz for both conditions.

### ERD analysis

**Fig. 5:**
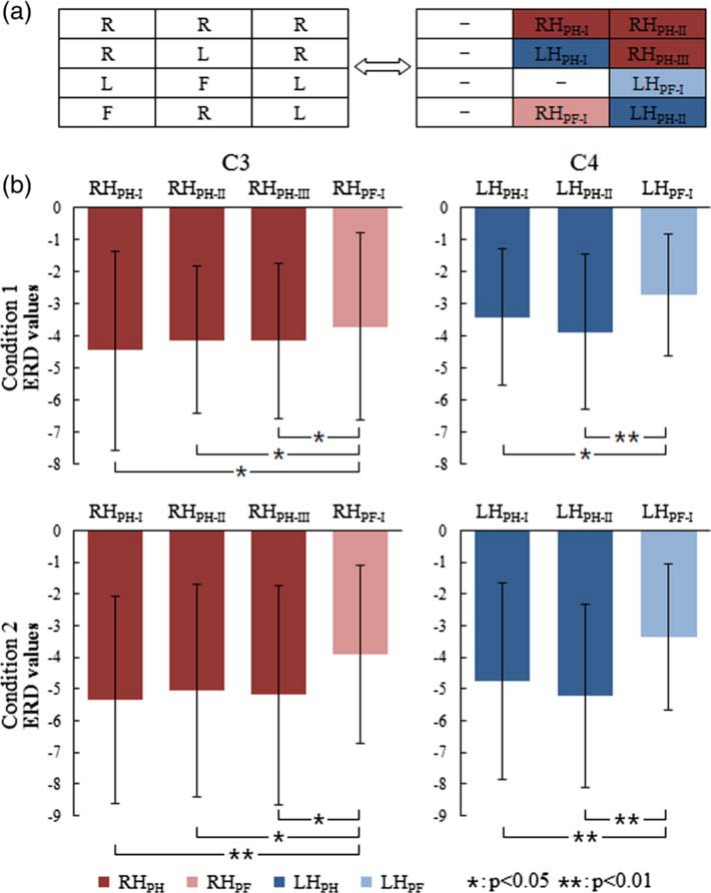
Illustration of ERD analysis. **a** Each row in the left diagram represents three sub-movements in each sequence. The diagram on the right contains the definition of the corresponding sub-movement in each sequence against the diagram on the left. “-” means no definition. **b** The comparison of ERD values from the 12 subjects at electrodes C3 (left column) and C4 (right column) for the first condition (upper panel) and second condition (lower panel). Dark red represents right hand imagery with prior hand imagery, while light red represents right hand imagery with prior foot imagery. Dark blue represents left hand imagery with prior hand imagery, while light blue represents left hand imagery with prior foot imagery. Conditions 1 and 2 indicate that the time intervals are 1 s and 2 s, respectively, for sub-movement imagination. Condition pairs that significantly differ from each other are indicated by a single asterisk (*p* < 0.05) or two asterisks (*p* < 0.01)

The ERD values within the alpha band during imagination of the second or third sub-movement were measured to evaluate the influence of the prior sub-movement on the subsequent sub-movement. According to Fig. 5a, right hand imagery with prior hand (left or right)/foot imagery is represented by RH_PH_/RH_PF_, while left hand imagery with prior hand (left or right)/foot imagery is represented by LH_PH_/LH_PF_. Therefore, with respect to the sub-movement of the right hand, we can obtain the ERD values of RH_PH-I_, RH_PH-II_, RH_PH-III_, and RH_PF-I_, while the ERD values of LH_PH-I_, LH_PH-II_, and LH_PF-I_ can be obtained for the sub-movement of the left hand. Paired *t*-test was used to inspect the difference between the ERD values. The results of the ERD values at electrodes C3 and C4 for both conditions are shown in Fig. 5b. The ERD values of RH_PH-I_, RH_PH-II_, and RH_PH-III_ are significantly larger than those of RH_PF-I_ at electrode C3, while the ERD values of LH_PH-I_ and LH_PH-II_ are significantly larger than those of LH_PF-I_ at electrode C4 for both conditions. These results indicate that the ERD values of left/right hand imagery with prior hand imagery are significantly larger than those with prior foot imagery during sequential movement imagination. For both conditions, there is no difference between the ERD values of RH_PH-I_, RH_PH-II_, and RH_PH-III_ at electrode C3; there is also no difference between the ERD values of LH_PH-I_ and LH_PH-II_ at electrode C4, indicating that there is no difference between the ERD values of hand imagery with prior left or right hand imagery during sequential movement imagination.

### PLV analysis

**Fig. 6:**
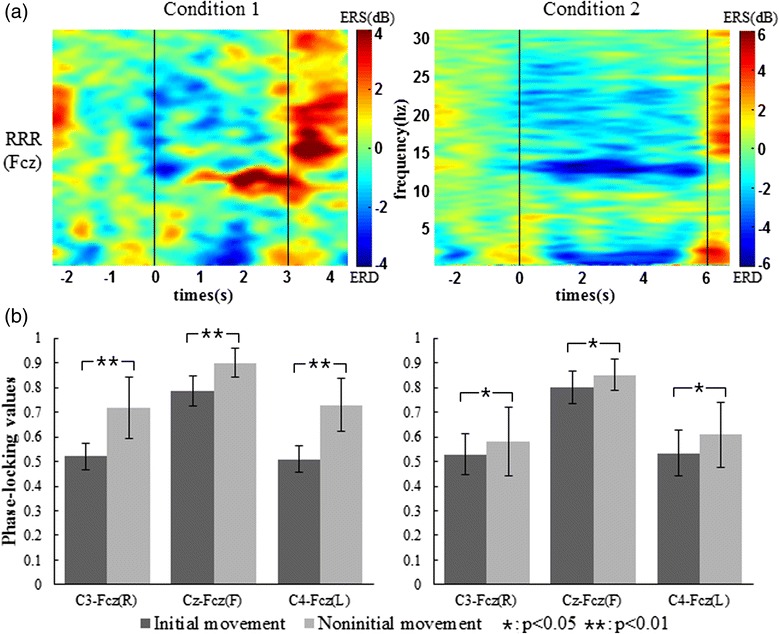
The time-frequency maps at electrode position Fcz during imagination of RRR from subject S8 (**a**) and the comparison of PLVs over C3-Fcz, Cz-Fcz, and C4-Fcz from the 12 subjects (**b**) for the first condition (left column) and second condition (right column). Conditions 1 and 2 indicate that the time intervals are 1 s and 2 s, respectively, for sub-movement imagination. RRR indicates [right hand]–[right hand]–[right hand]. Blue represents ERD and red represents ERS. The first and second vertical lines represent the onset and end of MI, respectively. Time 0 represents the time point of cue offset. C3-Fcz(R), Cz-Fcz(F), and C4-Fcz(L) indicate calculating the PLVs over C3-Fcz, Cz-Fcz, and C4-Fcz for the right hand, right foot, and left hand imagery during sequences, respectively. The black and gray bars represent initial and non-initial movements, respectively. Condition pairs that significantly differ from each other are indicated by an asterisk (*p* < 0.05) or two asterisks (*p* < 0.01)

Phase synchronization was used to evaluate the involvement of the mesial frontocentral cortex for each sub-movement imagination during sequential movement imagination. While analyzing PLVs for each sub-movement imagination in all sequences, we found the differences of PLVs between the initial movement (the first sub-movement) and the non-initial movement (the second or third sub-movement). In the following analysis, sub-movements in each sequence were divided into two groups: initial and non-initial movements. For right hand imagery, the initial movement consists of two initial imaginations during the sequence imagination of RRR and RLR, while the non-initial movement consists of four non-initial imaginations during the sequence imagination of RRR, RLR, and FRL. Similarly, the initial and non-initial imaginations of the left hand were integrated as a group, respectively. A homogeneity test of variance and the following two independent sample *t*-test were employed to investigate the differences of PLVs between the initial and non-initial movement imaginations for both conditions. As shown in Fig. 6a, ERD patterns around 13 Hz could be found at electrode Fcz during the imagination of RRR for both conditions. Figure 6b shows the comparative results of PLVs over C3-Fcz, Cz-Fcz, and C4-Fcz for both conditions. The PLVs over C3-Fcz during non-initial imaginations are significantly larger than those during initial imaginations for the right hand in both conditions. Meanwhile, the PLVs over Cz-Fcz for right foot imagery and the PLVs over C4-Fcz for left hand imagery present the same characteristic. The above results indicate that the PLVs over corresponding electrode pairs of the non-initial movement are significantly larger than those of the initial movement.

### Classification performance

**Fig. 7:**
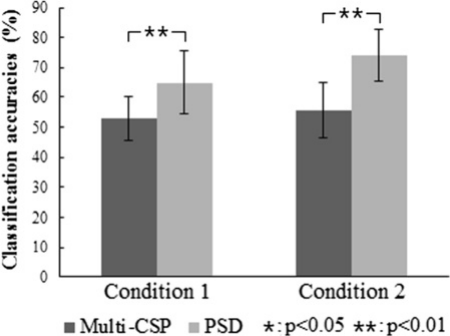
The classification accuracies of multi-CSP-based and PSD-based methods for both conditions from the 12 subjects. Conditions 1 and 2 indicate that the time intervals are 1 s and 2 s, respectively, for sub-movement imagination. The black and gray bars indicate the classification accuracies of the multi-CSP-based and the PSD-based methods, respectively. Condition pairs that significantly differ from each other are indicated by a single asterisk (*p* < 0.05) or two asterisks (*p* < 0.01)

To validate the separability of the four types of sequential movement imagination in this study, we used multi-CSP-based and PSD-based methods, and then analyzed the highest classification accuracies with optimal combination in each method for both conditions. The highest classification accuracies were obtained with optimal filter combination using the 64-channel EEG data in the multi-CSP-based method while those were obtained with optimal channel combination in the PSD-based method. Figure 7 illustrates the classification accuracies of multi-CSP-based and PSD-based methods for both conditions. The classification accuracies of the PSD-based method are significantly higher than those of the multi-CSP-based method, whose mean classification accuracies are lower than 60 % for both conditions.

**Fig. 8:**
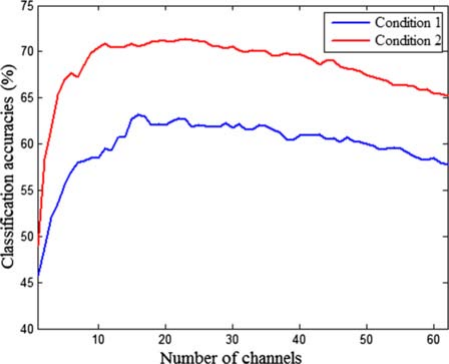
The average result of SVM-RFE in PSD-based method from the 12 subjects. The horizontal and vertical axes represent the number of channels selected and the classification accuracies, respectively. The blue and red lines represent the first and second conditions, respectively. Conditions 1 and 2 indicate that the time intervals are 1 s and 2 s, respectively, for sub-movement imagination

The averaged results developed over channels selected with SVM-RFE in the PSD-based method for both conditions are presented in Fig. 8. Results for both conditions show a similar trend reaching a maximum around 20 channels, and starting to decrease with more than 30 channels. The highest classification accuracy could be obtained with small channel subsets for both conditions, indicating a sharp decrease in channel numbers after feature optimization. Meanwhile, the classification accuracies of the second condition are higher than those of the first condition.

Table 1 shows the classification accuracies of the four MI tasks using the PSD-based method in both conditions for the 12 subjects. The highest accuracies are 82.86 % and 91.43 %, and the mean values are 65 % and 74.14 % for the first and second conditions, respectively. The classification accuracies of the second condition are significantly higher than those of the first condition, exceeded by ~9 % in the mean classification accuracy, at the 5 % significance level using *t*-test (*p* = 0.001).

**Table 1:** Classification accuracies (%) of the PSD-based method in both conditions from the 12 subjects

PSD-based method	S1	S2	S3	S4	S5	S6	S7	S8	S9	S10	S11	S12	mean
Condition 1	63.57	69.29	72.86	43.57	73.57	73.57	56.43	**82.86**	62.50	61.79	67.14	52.86	65.00
Condition 2	78.21	76.43	73.93	68.21	70.36	80.71	76.07	**91.43**	70.71	75.36	73.21	55.00	74.14

Conditions 1 and 2 indicate that the time intervals are 1 s and 2 s, respectively, for sub-movement imagination. The highest accuracy for each condition is in bold font

## Discussion

One disadvantage of MI-based BCI is the limitation of control commands. Several previous studies have been devoted to expanding the instruction set of MI-based BCI using simultaneous imagination of different limbs [17, 22, 28]. Although MI-based BCI has been frequently employed in rehabilitation engineering [29–31], rehabilitation training using MI-based BCI with sequential and multiple limbs involved in movement could be a trend in the future. In terms of SCL-MI, enough time should be ensured to complete each sub-movement imagination considering limb switching between different limbs. Moreover, information transfer rate (ITR) is also an important aspect to be considered for a BCI system. Therefore, we set two task conditions with two different time intervals in this study. Although it is difficult to compress the time needed during SCL-MI, there are more candidate instructions (except those used in this study) that could be employed in MI-based BCI systems as compared with simultaneous compound limb MI.

The results in time-frequency maps mainly show the difference of ERD changes between two conditions brought by the changes in time interval during SCL-MI. From the time-frequency maps of the second condition, the ERD patterns of sub-movements imagination were found clearly occurring within corresponding time intervals over time at electrodes C3 and C4, due to the characteristic of contralateral dominance with hand imagery [32]. Such a phenomenon indicates that the changes of ERD patterns over time are in line with the changes of limbs during SCL-MI. However, the changes of ERD patterns in the first condition are less obvious than those for the second condition during SCL-MI. One explanation could be that a time interval of 1 s is not enough for EEG power returning to baseline after sub-movement imagination. At the same time, with respect to the sequences of RLR and FRL, it may also result from the mixed effect from the prior hand imagery and subsequent hand imagery on the same electrode. The time-frequency results reflect the importance of time-parameter setting for the imagination of each sub-movement. On the other hand, no ERD phenomenon was observed in the foot area during sub-movement imagination of the right foot, which may be due to its special location in the mesial wall.

In terms of the inter-influence between sub-movements, the ERD analysis reflects that different prior sub-movement imaginations contribute to different influences on the subsequent sub-movement imagination. The statistical analysis reveals significantly larger ERD values of left/right hand imagery with prior hand imagery, indicating that the prior hand imagery results in more neurons activated during the subsequent hand imagery because an increased ERD represents the involvement of more cell assemblies in information processing [32]. In addition, we found that there is no significant difference in ERD values at electrode C3 between the first and second sub-movements, and between the second and third sub-movements during sequence imagination of RRR for both conditions. In this regard, the main effect of the prior sub-movement may be caused by movement type. However, the MI in this study is based on drum movements where hand movement is more prominent than foot movement. Probably, a dominant movement could facilitate or a non-dominant movement could inhibit the activation of contralateral hand area with the subsequent hand imagery during sequential movement imagination. Hence, we still need further studies where both hands and foot imagery have equal priority or foot imagery is more prominent than hand imagery to justify the above speculation. Moreover, the process switching from hand imagery to hand imagery seems to be more efficient than switching from foot imagery to hand imagery in the four motor sequences of the present study as more efficient task performance shows larger alpha desynchronization in semantic tasks [32, 33]. On the other hand, ERD patterns were observed at electrode Fcz over the mesial frontocentral cortex, suggesting that the mesial frontocentral cortex was activated during sub-movement imagination because ERD reflects the activity in motor areas during MI [32]. Different from the source analysis, functional connectivity could reveal mutual interaction among multiple cortical regions. As one method to calculate functional connectivity, phase synchronization has been measured between electrodes over the contralateral hand area and the mesial frontocentral cortex during executed and imagined hand movements [34, 35], suggesting that the mesial frontocentral cortex is activated and synchronized with the contralateral hand area. Here, we analyzed phase synchronization to evaluate the involvement of the mesial frontocentral cortex depending on the coupling degree between the corresponding electrodes for each sub-movement during SCL-MI. Compared with initial movement, a significantly larger PLV reveals a closer coupling relationship between central electrodes and the mesial frontocentral electrode for non-initial movement, which probably implies a higher involvement of the mesial frontocentral cortex during the information processing of non-initial movement imagination. It is inferred that the coupling degree between the hand/foot representation areas and the mesial frontocentral cortex may be at a lower level during the initiation of sequence movement imagination, and then gradually becomes strengthened during the subsequent sub-movement imagination. In terms of non-initial movement, the mesial frontocentral cortex has been activated during the prior sub-movement imagination so that it may be easier to be aroused during the subsequent sub-movement imagination. Probably, this is the reason for stronger phase synchronization during non-initial movement imagination. The results of PLV analysis also reflect the inter-influence between sub-movements, in particular, that prior sub-movement may facilitate the involvement of the mesial frontocentral cortex for the subsequent sub-movement during the whole period of sequential movement imagination. As a result, not only the neural activities of sensorimotor areas but also those of the mesial frontocentral cortex could be influenced during limb switching, indicating that the mental changes associated with the imagination of sub-movement in the motor sequence are different from those of simple limb MI with the same limb.

Considering the different frequency bands used in the two classification methods, we also computed and compared the classification accuracies within the same frequency band. The mean classification accuracy is 39.46 % (SD ± 6.13) for the first condition and 42.50 % (SD ± 8.98) for the second condition in 0.5–50 Hz using the multi-CSP-based method. The mean classification accuracy is 63.17 % (SD ± 10.36) for the first condition and 70.39 % (SD ± 9.87) for the second condition in 8–30 Hz using the PSD-based method. The classification accuracies in 8–30 Hz are better than those in 0.5–50 Hz for the multi-CSP-based method. The frequency band 8–30 Hz is chosen because it encompasses the alpha and beta frequency bands, which have been shown to be most important for movement classification [26]. However, besides alpha and beta bands, other frequency bands could also be helpful in the classification. For the PSD-based method, the classification accuracies in 0.5–50 Hz are better than those in 8–30 Hz. A broader band (0.5–50 Hz) could be used to select the optimal feature from a high-dimensional feature space using SVM-RFE in the PSD-based method. It is implied that the selection of the frequency bands for both methods in this paper is available to extract features in the classification of four kinds of SCL-MI. In the meantime, the performance of the PSD-based method outperformed that of the multi-CSP-based method in both frequency bands. CSP is an algorithm based on spatial filtering of raw signals, while the PSD-based method extracts EEG features over time. It can be inferred that the changes of temporal effect are stronger than those of spatial effect during SCL-MI. SCL-MI presents time-variant EEG patterns on frequency and spatial domains due to limb switching. As a result, multi-CSP that is insensitive to temporal effect seems to be inappropriate for the classification of SCL-MI, while the PSD-based method is more suitable for capturing time-variant information and for discriminating the four types of sequential movement imagination. In addition, the classification results of multi-CSP-based and PSD-based methods should serve as a guide for the next development of algorithms applied for SCL-MI in the future.

For SVM-RFE, after a given point in the feature selection loop, all useless or redundant channels have already been removed, and the algorithm begins to eliminate channels that carry nonredundant information [36]. Therefore, the decrease in classification accuracies when evaluating smaller subsets with less than ~20 channels is typical of feature selection methods. SVM-RFE selects small feature subsets (optimal channel combination) with better discrimination capabilities than using a whole feature set, indicating that SVM-RFE is an effective method for reducing feature dimension to reach the highest accuracy with the least channels. From the higher classification accuracies obtained in the second condition compared with those in the first condition, we can infer that SCL-MI presents much richer information of time-variant EEG patterns for the second condition. A time interval of 2 s is enough for the changes of EEG patterns during limb switching just as the ERD changes revealed in time-frequency maps. The four sequential MI tasks could not be discriminated effectively with the smaller time interval, which also indicates the significance of the time required for sub-movement imagination. Overall, the classification accuracies in this study are acceptable, but have yet to be improved with more suitable algorithms in the future. In conclusion, multi-class SCL-MI and the feature extraction method studied in this paper could be expected to provide technical support to expand instructions of MI-based BCI systems effectively. Furthermore, as an important tool in the field of rehabilitation engineering, it would seem significant to consider the tendency and availability to complete sequences as a whole rather than one independent movement at a time in MI-based BCI systems.

## Conclusion

This study provides new information about ERD changes with different time intervals during SCL-MI. Aside from this finding, prior sub-movement imagination could affect the neural activities in the motor areas during the subsequent sub-movement imagination, suggesting that inter-influence exists between sub-movements in the motor sequence. On the basis of classification performance, multiple types of sequential movement imagination appear to have potential for use as a novel paradigm in multi-class MI-based BCI system.
